# Supplementary material for: Assessing the best time interval between doses in a two-dose vaccination regimen to reduce the number of deaths in an ongoing epidemic of SARS-CoV-2
Source: PLoS Comput Biol. 2022 Mar 25;18(3):e1009978. doi: 10.1371/journal.pcbi.1009978 (PMC8986122; doi:10.1371/journal.pcbi.1009978)
Supplement: S1 Text — The accompanying file contains the mathematical description of the model, together with the epidemiological parameters used in the simulations. (PDF) [file pcbi.1009978.s001.pdf]

Supplementary Material – Assessing the best time interval between doses in a two-dose vaccination regimen to reduce the number of deaths in an ongoing epidemic of SARS-CoV-2

Leonardo Souto Ferreira[1,2], Otavio Canton[1,2], Rafael Lopes Paixão da Silva[1,2], Silas Poloni[1,2], Vítor Sudbrack[1,2,3], Marcelo Eduardo Borges[2,4], Caroline Franco[1,2,5], Flavia Maria Darcie Marquitti[2,6], José Cássio de Moraes[2,7], Maria Amélia de Sousa Mascena Veras[2,7], Roberto André Kraenkel[1,2], and Renato Mendes Coutinho[2,4]

[1]*Instituto de Física Teórica, Universidade Estadual Paulista, São Paulo, Brazil*
[2]*Observatório Covid-19 BR, São Paulo, Brazil*
[3]*Department of Ecology and Evolution, University of Lausanne, Lausanne, Switzerland*
[4]*Centro de Matemática, Computação e Cognição, Universidade Federal do ABC, Santo André, Brazil*
[5]*Big Data Institute, Li Ka Shing Centre for Health Information and Discovery, Nuffield Department of Medicine, University of Oxford, Oxford, UK*
[6]*Instituto de Física 'Gleb Wataghin' and Instituto de Biologia, Universidade Estadual de Campinas, Campinas, Brazil*
[7]*Faculdade de Ciências Médicas da Santa Casa de São Paulo, São Paulo, Brazil*

I. Introduction

This supplementary material describes more thoroughly our model. In section II We have the equations that describe the model. III contains the epidemiological parameters of the model (the vaccination parameters are described in the main text), together with our mathematical approach to vaccine efficacies in III-A. Finally, in section IV we describe the mathematical approach to calculate Rt and the initial conditions of the model.

II. Model Equations

To model the virus dispersal in the population, we assume that asymptomatic individuals have equal infectiousness compared to symptomatic ones, while pre-symptomatics have reduced infectiousness given by ω. To model behaviour, we assume that symptomatic individuals isolate themselves at some degree, reducing their contacts by ξ, individuals with severe disease have greater isolation ξ_{sev} due to hospitalization. The daily contacts between each age class is given by the matrix $\hat{C}$ and the force of infection λ is given below:

$$\begin{aligned}\lambda = \hat{C}[&A + \omega E + (1-\xi)I + (1-\xi_{sev})H + \\ &A_v + \omega E_v + (1-\xi)I_v + (1-\xi_{sev})H_v + \\ &A_w + \omega E_w + (1-\xi)I_w + (1-\xi_{sev})H_w]\end{aligned} \tag{1}$$

Our model does not assume a reduction in infectiousness by vaccination given the lack of data[1].

[1]We expect that this would not change the results qualitatively.

Unvaccinated

$$\frac{dS}{dt} = -\beta\lambda\frac{S}{N} - v(t)\frac{S}{S+R} \tag{2a}$$
$$\frac{dE}{dt} = \beta\lambda\frac{S}{N} - \frac{E}{\gamma} \tag{2b}$$
$$\frac{dA}{dt} = \frac{\alpha(1-\sigma)E}{\gamma} - \frac{A}{\nu_i} \tag{2c}$$
$$\frac{dI}{dt} = \frac{(1-\alpha)(1-\sigma)E}{\gamma} - \frac{I}{\nu_i} \tag{2d}$$
$$\frac{dH}{dt} = \frac{\sigma E}{\gamma} - \frac{H}{\nu_s} \tag{2e}$$
$$\frac{dR}{dt} = \frac{A}{\nu_i} + \frac{I}{\nu_i} + \frac{(1-\mu)H}{\nu_s} - v(t)\frac{R}{S+R} \tag{2f}$$
$$\frac{dD}{dt} = \frac{\mu H}{\nu_s} \tag{2g}$$

Vaccinated once

$$\frac{dS_v}{dt} = -\beta_v\lambda\frac{S_v}{N} + v(t)\frac{S}{S+R} - (1-\theta)v(t-a)\frac{S(t-a)}{S(t-a)+R(t-a)} \tag{2h}$$
$$\frac{dE_v}{dt} = \beta_v\lambda\frac{S_v}{N} - \frac{E_v}{\gamma} \tag{2i}$$
$$\frac{dA_v}{dt} = \frac{\alpha_v(1-\sigma_v)E_v}{\gamma} - \frac{A_v}{\nu_i} \tag{2j}$$
$$\frac{dI_v}{dt} = \frac{(1-\alpha_v)(1-\sigma_v)E_v}{\gamma} - \frac{I_v}{\nu_i} \tag{2k}$$
$$\frac{dH_v}{dt} = \frac{\sigma_v E_v}{\gamma} - \frac{H_v}{\nu_s} \tag{2l}$$
$$\frac{dR_v}{dt} = \frac{A_v}{\nu_i} + \frac{I_v}{\nu_i} + \frac{(1-\mu_v)H_v}{\nu_s} + v(t)\frac{R}{S+R} - (1-\theta)v(t-a)\frac{R(t-a)}{S(t-a)+R(t-a)} \tag{2m}$$
$$\frac{dD_v}{dt} = \frac{\mu_v H_v}{\nu_s} \tag{2n}$$

Vaccinated twice

$$\frac{dS_w}{dt} = -\beta_w\lambda\frac{S_w}{N} + (1-\theta)v(t-a)\frac{S(t-a)}{S(t-a)+R(t-a)} \tag{2o}$$
$$\frac{dE_w}{dt} = \beta_w\lambda\frac{S_w}{N} - \frac{E_v}{\gamma} \tag{2p}$$
$$\frac{dA_w}{dt} = \frac{\alpha_w(1-\sigma_w)E_w}{\gamma} - \frac{A_w}{\nu_i} \tag{2q}$$
$$\frac{dI_w}{dt} = \frac{(1-\alpha_w)(1-\sigma_w)E_w}{\gamma} - \frac{I_w}{\nu_i} \tag{2r}$$
$$\frac{dH_w}{dt} = \frac{\sigma_w E_w}{\gamma} - \frac{H_w}{\nu_s} \tag{2s}$$
$$\frac{dR_w}{dt} = \frac{A_w}{\nu_i} + \frac{I_w}{\nu_i} + \frac{(1-\mu_w)H_w}{\nu_s} + (1-\theta)v(t-a)\frac{R(t-a)}{S(t-a)+R(t-a)} \tag{2t}$$
$$\frac{dD_w}{dt} = \frac{\mu_w H_w}{\nu_s} \tag{2u}$$

The equations were numerically solved by the R package developed by FitzJohn and Hinsley [3].

III. Parameterization of the model

The parameters that do not depend on vaccination are given in Table I.

TABLE I: Epidemiological parameters

Parameter	Description	Value	Source
γ	Average time in days between being infected and developing symptoms	5.8	Wei, Wei, Liu, et al. [10]
ν_i	Average time in days between being infectious and recovering for asymptomatic and mild cases	9.0	Cevik, Tate, Lloyd, et al. [2]
ν_s	Average time between being infectious and recovering/dying for severe cases	8.4	SIVEP-Gripe for São Paulo State [4]
ξ	Reduction on the exposure of symptomatic cases (due to symptoms/quarantining)	0.1	Assumed
ξ_{sev}	Reduction on the exposure of severe cases (due to hospitalization)	0.9	Assumed
ω	Relative infectiousness of pre-symptomatic individuals	1.0	Assumed
α	Proportion of asymptomatic cases	[0.67,0.44,0.31]	Juvenile [8]<br>Adult and Elderly [9]
σ	Proportion of infectious cases that require hospitalization	[0.001, 0.014, 0.099]	Salje, Kiem, Lefrancq, et al. [7]
μ	In-hospital mortality ratio	[0.417,0.188,0.754]	Portella, Mortara, Lopes, et al. [5]

A. *Efficacy parameters computation from observed efficacies*

The vaccinated classes parameters are combined with vaccine efficacies as:

$$\begin{aligned} \beta_v &= (1-\epsilon_{\beta,v})\beta & \alpha_v &= 1-(1-\epsilon_{\alpha,v})(1-\alpha) \\ \beta_w &= (1-\epsilon_{\beta,w})\beta & \alpha_w &= 1-(1-\epsilon_{\alpha,w})(1-\alpha) \\ \sigma_v &= (1-\epsilon_{\sigma,v})\sigma & \mu_v &= (1-\epsilon_{\mu,v})\mu \\ \sigma_w &= (1-\epsilon_{\sigma,w})\sigma & \mu_w &= (1-\epsilon_{\mu,w})\mu \end{aligned} \tag{3}$$

To avoid multiplicative effects in vaccine efficacies, we need to calculate the efficacy parameters from the reported values. Let us start with the risk of infection. In our model, this is given by β. Thus the observed efficacy against infection E_β is given by:

$$E_\beta = 1 - \frac{(1-\epsilon_\beta)\beta}{\beta} = \epsilon_\beta \tag{4}$$

Therefore the protection against infection parameter is simply the observed efficacy. Note that we dropped the dose index as these expressions are valid for both first and second dose efficacies.

The risk of individuals being hospitalized is given by $\beta\sigma$, therefore, the observed efficacy in reducing hospitalized cases E_σ is then given by:

$$E_\sigma = 1 - \frac{(1-\epsilon_\beta)\beta(1-\epsilon_\sigma)\sigma}{\beta\sigma} = 1-(1-\epsilon_\beta)(1-\epsilon_\sigma) \tag{5}$$

In terms of known values, the protection against hospitalization is given by:

$$\epsilon_\sigma = 1 - \frac{1-E_\sigma}{1-E_\beta} \tag{6}$$

Being μ the proportion of hospitalized individuals that die, we have that the risk of an individual being infected and die is given by $\beta\sigma\mu$, therefore the observed efficacy against death E_μ is given by:

$$E_\mu = 1 - \frac{(1-\epsilon_\beta)\beta(1-\epsilon_\sigma)\sigma(1-\epsilon_\mu)\mu}{\beta\sigma\mu} = 1-(1-\epsilon_\beta)(1-\epsilon_\sigma)(1-\epsilon_\mu) \tag{7}$$

We then can obtain ε_μ in terms of known values:

$$\varepsilon_\mu = 1 - \frac{1-E_\mu}{(1-\epsilon_\beta)(1-\epsilon_\sigma)} = 1 - \frac{1-E_\mu}{(1-E_\beta)\frac{(1-E_\sigma)}{(1-E_\beta)}} = 1 - \frac{1-E_\mu}{1-E_\sigma} \tag{8}$$

In our model, symptomatic cases are given by severe (hospitalized) and mild cases, the risk of becoming a symptomatic individual is given by $\beta[\sigma+(1-\sigma)(1-\alpha)]$, then the observed efficacy E_{symp} is given by:

$$E_{symp} = 1 - \frac{(1-\epsilon_\beta)\beta\{(1-\varepsilon_\sigma)\sigma + [1-(1-\varepsilon_\sigma)\sigma][(1-\varepsilon_\alpha)(1-\alpha)]\}}{\beta[\sigma+(1-\sigma)(1-\alpha)]} \tag{9}$$

Thus
$$\frac{(1-E_{symp})[\sigma+(1-\sigma)(1-\alpha)]}{1-\epsilon_\beta}=(1-\epsilon_\sigma)\sigma+[1-(1-\epsilon_\sigma)\sigma][(1-\epsilon_\alpha)(1-\alpha)] \tag{10}$$

Then
$$1-\epsilon_\alpha=\frac{\frac{(1-E_{symp})}{(1-\epsilon_\beta)}[\sigma+(1-\sigma)(1-\alpha)]-(1-\epsilon_\sigma)\sigma}{[1-(1-\epsilon_\sigma)\sigma](1-\alpha)} \tag{11}$$

Therefore, ε_α is given in terms of known variables as:
$$\varepsilon_\alpha=1-\frac{(1-E_{symp})[\sigma+(1-\sigma)(1-\alpha)]-(1-E_\sigma)\sigma}{(1-E_\beta)\left[1-\frac{(1-E_\sigma)}{(1-E_\beta)}\sigma\right](1-\alpha)} \tag{12}$$

Note that $1-E_{symp}$ does not multiply the whole expression.

IV. Effective reproduction number and initial conditions estimation

Both initial conditions estimation and effective reproduction number calculations go through rewriting the model in a different notation. It is a system of equations for two different groups, infected ($\mathbf{y}$) and non-infected ($\mathbf{z}$) populations, being $\mathbf{y}=(E,\ A,\ I,\ H)^T$, and $\mathbf{z}=(S,\ R,\ D)^T$. Note that none of the vaccinated classes are considered since at the initial condition no vaccine has been applied yet.

We write the system

$$\begin{aligned}\dot{\mathbf{y}} &= F(\mathbf{y},\mathbf{z})-G(\mathbf{y},\mathbf{z}), &(13)\\ \dot{\mathbf{z}} &= J(\mathbf{y},\mathbf{z})\ , &(14)\end{aligned}$$

where F are all entries of new Infected, coming from classes $\mathbf{z}$, whilst G accounts for the transitions within infected classes and also recovery and death from the disease. J accounts for the exits of the susceptible population to exposed classes, and the entrance of recovered and deceased in their respective compartments. Consider a linearization around a fixed vector $\mathbf{z}=\tilde{\mathbf{z}}$, the equation for $\mathbf{y}$ becomes

$$\dot{\mathbf{y}}=(\hat{F}-\hat{G})\mathbf{y}\ , \tag{15}$$

where $\hat{F}$ and $\hat{G}$ are matrices that appear from linearizing functions F and G, respectively. Remembering that each of the compartments is divided into three age sub-compartments, that is $\mathbf{S}=(S_{young},S_{adult},S_{elderly})$ and that the only entrance of new infected comes from the $\beta\mathbf{S}\lambda/N$ terms in the $\dot{E}$ equations, we write

$$\hat{F}=\frac{\beta}{N}\begin{bmatrix}\omega\hat{b} & \hat{b} & (1-\xi)\hat{b} & (1-\xi_{sev})\hat{b}\\ & \multicolumn{2}{c}{\mathbb{0}_{9,12}} & \end{bmatrix}\ , \tag{16}$$

where
$$\hat{b}=\mathrm{diag}(\mathbf{S})\hat{C}\ , \tag{17}$$

being $\hat{C}$ the contact matrices, from [6], ω the relative infectiousness of exposed individuals and ξ and ξ_{sev} the reductions in contacts of people that are symptomatic and hospitalized, respectively.

Now, $\hat{G}$ contains the terms of Exposed, E, developing the possible forms of disease considered in the model as the terms in its first 3 rows, while it's main diagonal contains terms of recovery and death, writing

$$\hat{G}=\begin{bmatrix}\gamma^{-1} & \mathbb{0} & \mathbb{0} & \mathbb{0}\\ -\alpha(1-\sigma)\gamma^{-1} & \nu_i^{-1} & \mathbb{0} & \mathbb{0}\\ -(1-\alpha)(1-\sigma)\gamma^{-1} & \mathbb{0} & \nu_i^{-1} & \mathbb{0}\\ -\sigma\gamma^{-1} & \mathbb{0} & \mathbb{0} & \nu_s^{-1}\end{bmatrix}. \tag{18}$$

Now, F and G are important for both R_t calculation and initial conditions estimation. Note that for the linear problem, assuming, $\mathbf{y}(t)=\sum_i \mathbf{a}_i e^{r_i t}$, $\mathbf{a}_i$ constant vectors, yields

$$\sum_i r_i\mathbf{a}_i e^{r_i t}=(\hat{F}-\hat{G})\sum_i \mathbf{a}_i e^{r_i t}. \tag{19}$$

Defining $r^* = \max r_i$, $\mathbf{a}^*$ the vector associated with the exponential coefficient r^*, and dividing the above equation by $e^{r^* t}$, we get

$$\sum_i r_i \mathbf{a}_i e^{(r_i - r^*)t} = (\hat{F} - \hat{G}) \sum_i \mathbf{a}_i e^{(r_i - r^*)t} \ , \tag{20}$$

so after some time t elapses, the tuple $(\mathbf{a}^*, r^*)$ dominate the dynamics, and we're left with

$$r^* \mathbf{a}^* = (\hat{F} - \hat{G})\mathbf{a}^* \tag{21}$$

The main eigenvector of $\hat{F} - \hat{G}$, $\mathbf{a}^*$, gives a distribution of infected individuals among different classes. With hospitalizations per day data, we can fit a re-scaling factor for the eigenvector to match the term of hospital entrances ($\sigma\gamma^{-1}E$).

Notably, the effective reproduction calculation can be performed with $\hat{F}$ and $\hat{G}$ as

$$R_t = \rho(FG^{-1}) \ , \tag{22}$$

where $\rho(FG^{-1})$ is the spectral radius of FG^{-1}, which may be seen as the dominant eigenvalue of FG^{-1} in the simplest cases. The derivation of said result can be checked in multiple textbooks, see, for instance, chapter 6 in Allen, Brauer, Van den Driessche, et al. [1].

References

[1] Linda JS Allen, Fred Brauer, Pauline Van den Driessche, et al. *Mathematical epidemiology*. Vol. 1945. Springer, 2008.

[2] Muge Cevik, Matthew Tate, Ollie Lloyd, et al. "SARS-CoV-2, SARS-CoV, and MERS-CoV viral load dynamics, duration of viral shedding, and infectiousness: a systematic review and meta-analysis". In: *The Lancet Microbe* 2.1 (Jan. 2021), e13–e22. ISSN: 26665247. DOI: 10.1016/S2666-5247(20)30172-5. URL: https://linkinghub.elsevier.com/retrieve/pii/S2666524720301725.

[3] Rich FitzJohn and Wes Hinsley. *dde: Solve Delay Differential Equations*. R package version 1.0.1. 2020. URL: https://CRAN.R-project.org/package=dde.

[4] MS. *SRAG 2021 - Banco de Dados de Síndrome Respiratória Aguda Grave - incluindo dados da COVID-19*. https://opendatasus.saude.gov.br/dataset/bd-srag-2021, [Accessed: 2021-07-28]. 2021.

[5] Tatiana Pineda Portella, Sara Ribeiro Mortara, Rafael Lopes, et al. "Temporal and geographical variation of COVID-19 in-hospital fatality rate in Brazil". In: *medRxiv* (Feb. 2021). DOI: 10.1101/2021.02.19.21251949. URL: https://doi.org/10.1101/2021.02.19.21251949.

[6] Kiesha Prem, Kevin van Zandvoort, Petra Klepac, et al. "Projecting contact matrices in 177 geographical regions: An update and comparison with empirical data for the COVID-19 era". In: *PLOS Computational Biology* 17.7 (July 2021). Ed. by Matthew (Matt) Ferrari, e1009098. DOI: 10.1371/journal.pcbi.1009098. URL: https://doi.org/10.1371/journal.pcbi.1009098.

[7] Henrik Salje, Cécile Tran Kiem, Noémie Lefrancq, et al. "Estimating the burden of SARS-CoV-2 in France". In: *Science* 369.6500 (May 2020), pp. 208–211. DOI: 10.1126/science.abc3517. URL: https://doi.org/10.1126/science.abc3517.

[8] Secretaria Municipal de Saúde - Município de São Paulo. *Inquérito sorológico para Sars-Cov-2: Prevalência da infecção em escolares das redes públicas e privada da cidade de São Paulo*. http://www.capital.sp.gov.br/arquivos/pdf/2021/coletiva_saude_14-01.pdf, [Accessed: 2021-01-31]. 2021.

[9] W. W. Sun, F. Ling, J. R. Pan, et al. "Epidemiological characteristics of COVID-19 family clustering in Zhejiang Province". In: *Chinese journal of preventive medicine* 54.6 (2020), pp. 625–629. ISSN: 02539624. DOI: 10.3760/cma.j.cn112150-20200227-00199.

[10] Yongyue Wei, Liangmin Wei, Yihan Liu, et al. "A systematic review and meta-analysis reveals long and dispersive incubation period of COVID-19". In: *medRxiv* (2020).
